# Neural Response during the Activation of the Attachment System in Patients with Borderline Personality Disorder: An fMRI Study

**DOI:** 10.3389/fnhum.2016.00389

**Published:** 2016-08-02

**Authors:** Anna Buchheim, Susanne Erk, Carol George, Horst Kächele, Philipp Martius, Dan Pokorny, Manfred Spitzer, Henrik Walter

**Affiliations:** ^1^Institute of Psychology, University of InnsbruckInnsbruck, Austria; ^2^Department of Psychiatry and Psychotherapy, Division of Mind and Brain Research, University Hospital CharitéBerlin, Germany; ^3^Department of Psychology, Mills CollegeOakland, CA, USA; ^4^International Psychoanalytic University BerlinBerlin, Germany; ^5^Psychosomatic Hospital, HöhenriedBerlin, Germany; ^6^Department of Psychosomatic Medicine and Psychotherapy, University of UlmUlm, Germany; ^7^Department of Psychiatry III, University of UlmUlm, Germany

**Keywords:** borderline personality disorder, emotional regulation, attachment system, amygdala, functional MRI

## Abstract

Individuals with borderline personality disorder (BPD) are characterized by emotional instability, impaired emotion regulation and unresolved attachment patterns associated with abusive childhood experiences. We investigated the neural response during the activation of the attachment system in BPD patients compared to healthy controls using functional magnetic resonance imaging (fMRI). Eleven female patients with BPD without posttraumatic stress disorder (PTSD) and 17 healthy female controls matched for age and education were telling stories in the scanner in response to the Adult Attachment Projective Picture System (AAP), an eight-picture set assessment of adult attachment. The picture set includes theoretically-derived attachment scenes, such as separation, death, threat and potential abuse. The picture presentation order is designed to gradually increase the activation of the attachment system. Each picture stimulus was presented for 2 min. Analyses examine group differences in attachment classifications and neural activation patterns over the course of the task. Unresolved attachment was associated with increasing amygdala activation over the course of the attachment task in patients as well as controls. Unresolved controls, but not patients, showed activation in the right dorsolateral prefrontal cortex (DLPFC) and the rostral cingulate zone (RCZ). We interpret this as a neural signature of BPD patients’ inability to exert top-down control under conditions of attachment distress. These findings point to possible neural mechanisms for underlying affective dysregulation in BPD in the context of attachment trauma and fear.

## Introduction

Disturbances in the processing and regulation of emotions are core symptoms of borderline personality disorder (BPD; Leichsenring et al., 2011; Herpertz and Bertsch, 2015; Schulze et al., 2016). Emotional dysregulation is thought to result from increased impulsivity combined with the inability to modulate emotional responses (Linehan et al., 1994; Skodol et al., 2002; Lieb et al., 2004; Putnam and Silk, 2005; Conklin et al., 2006; Viviani et al., 2011; Mitchell et al., 2014; Scherpiet et al., 2014; Schmahl et al., 2014; Herpertz and Bertsch, 2015; van Zutphen et al., 2015).

Attachment theory provides a powerful framework for understanding links between close relationships, mental representations of attachment, and psychopathology (Westen et al., 2006; Bakermans-Kranenburg and van IJzendoorn, 2009). Early experiences of maltreatment, such as sexual and physical abuse and emotional neglect, are implicated in the etiology of BPD (Bandelow et al., 2005; Gunderson et al., 2006; Zanarini et al., 2006; van Dijke et al., 2011; Keinänen et al., 2012; Frías et al., 2016). Adverse attachment experiences, especially relationships trauma, are considered to be risk factors for poor emotion-regulation, functional impairment of mentalization, separation anxiety, and fear (Bowlby, 1973; Milrod et al., 2014; Mosquera et al., 2014; Brüne et al., 2016). The concept of adult attachment concerns an individual’s current representational state with respect to early attachment relationships and their associated modes of defense and affect regulation. A large number of studies in adult attachment research have used the Adult Attachment Interview (AAI) or the Adult Attachment Projective Picture System (AAP) as narrative-based assessments of mental representations of attachment (George et al., 1984/1985/1996; Main and Goldwyn, 1995; George et al., 1999; George and West, 2001, 2012). Attachment classifications are derived from the analysis of narratives with three organized/resolved patterns (also termed attachment status), namely secure, dismissing, preoccupied and unresolved with respect to trauma or loss. Important for our study is the categorization into “resolved” (i.e., either secure, insecure dismissing, insecure-preoccupied) vs. “unresolved” attachment patterns in adults. The narratives of individuals who are unresolved demonstrate their inability to contain or integrate frightening thematic elements. These individuals, overwhelmed by trauma or loss, become dysregulated when attachment is activated during assessment. Dysregulation can be momentary or prolonged, but in either case the individual is unable to use defensive processes to remain organized and recover from conscious thoughts and feelings of frightening distress.

Insecure attachment patterns demonstrate risk for the maladaptive personality traits underlying BPD (Scott et al., 2009). BPD has been associated with increased occurrence of insecure and especially unresolved attachment representations (Agrawal et al., 2004; Bakermans-Kranenburg and van IJzendoorn, 2009; Buchheim and George, 2011). Unresolved attachment has been linked to psychological disorders, impaired cognitive functioning and trauma-related psychopathology (Fearon and Mansell, 2001; Nakash-Eisikovits et al., 2002; Lyons-Ruth and Jacobvitz, 2008; Joubert et al., 2012).

Emotional vulnerability in BPD patients may result from a marked sensitivity to emotional stimuli, an impairment of emotion regulation, or both (Gunderson and Lyons-Ruth, 2008). An attentional bias toward negative information in patients with BPD can be seen in neuroimaging data showing increased and prolonged amygdala responses (Herpertz et al., 2001; Donegan et al., 2003; Hazlett et al., 2012; Kamphausen et al., 2013; Schulze et al., 2016), and enhanced activity in the anterior insula (Schulze et al., 2011; Ruocco et al., 2013). Furthermore, study results indicate reductions in amygdala and hippocampal volume in BPD patients (Driessen et al., 2000; Schmahl et al., 2003, 2009; van Elst et al., 2003; Irle et al., 2005; O’Neill and Frodl, 2012; Rossi et al., 2012; O’Neill et al., 2013; Ruocco et al., 2013).

As emotion regulation is dependent on regions exerting cognitive control, like the dorsolateral prefrontal cortex (DLPFC) and medial prefrontal/anterior cingulate cortex (mPFC/ACC; for a review see Ochsner and Gross, 2005), a deficit in emotion regulation should be mediated by reduced activation in these regions in tasks affording emotion control. Studies using Positron emission tomography revealed hypometabolism in PFC of BPD patients compared with healthy controls (De La Fuente et al., 1997; Soloff et al., 2000) and enhanced activation of DLPFC when BPD patients were confronted with individual scripts that evoke personal memories of abandonment and abuse (Schmahl et al., 2003, 2004; Lang et al., 2012). In another study using magnetic resonance spectroscopy (van Elst et al., 2001), BPD patients showed a decreased level of N-acetyl aspartate, which suggests impaired neural functioning in the DLPFC. BPD patients showed metabolic alterations in the amygdala using proton magnetic resonance spectroscopy (Hoerst et al., 2010).

Moreover studies investigated brain activation during processing of autobiographical memory in BPD. One study examining unresolved as compared to resolved life events found, among other regions, increasing activation of amygdala and anterior cingulate cortex (Beblo et al., 2006). A follow up analysis from that study 1 year later reported substantial decrease of temporo-frontal activation during the recall of unresolved negative life, suggesting that these activations were not stable over time (Driessen et al., 2009). Mensebach et al. (2009), comparing episodic and semantic memory retrieval, demonstrated that BPD patients might need to engage larger brain areas to reach a level of performance in episodic and semantic retrieval tasks than comparable healthy controls. A recent study using a stimulus-driven Episodic Memory task showed that negative affective interference with cognitive processing differed in BPD patients compared to healthy controls (Soloff et al., 2015). This pattern was associated with functional abnormalities in brain networks reported to have structural or metabolic abnormalities, like the increased activation of the amygdala (Soloff et al., 2015).

In sum, emotion regulation and their neural correlates are impaired in BPD patients; for example, amygdala responses are prolonged and enhanced in particular during the presentation of emotional stimuli (for a review see Buchheim et al., 2013). Furthermore, traumatic experiences are considered as crucial in these processes. These impairments may be part of the neural mechanisms underlying emotional dysregulation in BPD patients (Kamphausen et al., 2013). Functional anomalies might also have an important impact on cognitive processes. A recent study of Enzi et al. (2013) demonstrated that impaired emotion processing seems to affect the reward system in patients with BPD. Moreover we can conclude, that patients with BPD have been shown to exhibit impaired neuronal activity in areas of the medial and lateral PFC that control and modulate emotional activation, thereby covering topdown processes. Functional neuroimaging studies examined prefrontal hypometabolism during regulatory control processes (O’Neill and Frodl, 2012), and a recent metaanalysis of functional magnetic resonance imaging (fMRI) studies across different stimulation procedures (Ruocco et al., 2013). These studies reported enhanced neuronal activity in the insula but reduced activity in the subgenual ACC and DLPFC in patients with BPD as compared with healthy subjects (O’Neill and Frodl, 2012; Ruocco et al., 2013).

The functional neuroimaging studies summarized above measured brain activation patterns in response to visual stimuli (pictures, faces) or passively presented scripts. Several studies have investigated the neural correlates of “social” attachment (i.e., defined loosely as individuals in intimate relationships) in healthy populations. The main paradigm in these studies is the presentation of pictures of the beloved sexual partner or their own infant by contrasting familiar vs. non-familiar stimuli (Bartels and Zeki, 2000, 2004; Leibenluft et al., 2004; Nitschke et al., 2004; Gillath et al., 2005; Coan et al., 2006; Lenzi et al., 2009; Riem et al., 2012; Wittfoth-Schardt et al., 2012). These studies have used various approaches to identify the neural correlates of distinctive attachment-related systems (caregiving or sexual system). Taking into account the differences in methods and aims, these studies have reported a common neural activity for the romantic and maternal attachment in regions associated with reward and motivation and affective processing. However, much less is known about the neural correlates of attachment representations, for example, when individuals are instructed to tell stories to attachment related pictures while being scanned in an fMRI environment.

Buchheim et al. (2006a,b) developed an fMRI paradigm to investigate the neural correlates of attachment representation while subjects tell stories to attachment pictures using the AAP stimuli (George et al., 1999; George and West, 2012). This validated narrative-based assessment is a set of stimuli and system of analysis that is theoretically derived using attachment theory, and not a traditional open-ended free-response measurement (i.e., subjective interpretation of emotional stimuli). The results of this pilot study showed unresolved attachment to be associated with increasing activation of the right amygdala, the left hippocampus and the right PFC over the course of the attachment task (i.e., from the first to the last attachment pictures).

The present study investigated neural correlates of attachment narratives in borderline patients using the same paradigm. In this article, we focus on differences between “unresolved” and “resolved” subjects. Our design permitted analysis related to other linguistic features of the AAP, which were reported elsewhere (Buchheim et al., 2008). Based on research linking BPD to unresolved attachment, we expected that amygdala activation in during the course of administering the attachment task (AAP) would be stronger in the patient group than the control group. We also expected that neural signs of emotion regulation in the cognitive control system (DLPFC and/or anterior cingulate) would decrease or be absent in the patient group.

## Materials and Methods

### Participants

Thirteen female BPD inpatients were recruited from a psychiatric hospital (Psychosomatic and Psychiatric Hospital, Bad Wiessee, Germany) and compared to 21 healthy female volunteers recruited for the study by an advertisement in a local newspaper and leaflets distributed in the Hospital of the University of Ulm. The sample of the 17 healthy controls in this study included the 11 subjects of our pilot study (Buchheim et al., 2006b). Subjects were matched for age and education. Patients were treated on an average for 114 days (SD: 45.3, range 26–166 days) in the psychiatric hospital and had on an average 1.77 days of inpatient stays (range 1–4, median 1). They were assessed at the beginning of their treatment. All control subjects were physically healthy, without a history of psychiatric disorder and did not use any medication. Clinical diagnoses were assessed by a trained psychiatrist^1^ using the Structured Clinical Interview for DSM-IV (SCID-I and SCID-II) and the International Personality Disorder Examination (IPDE). Exclusion criteria were left handedness, metal implants, language problems, and serious medical or neurological illness, including comorbid psychotic disorders and bipolar disorder. None of our patients met diagnostic criteria for posttraumatic stress disorder (PTSD) or dissociative disorder. Six subjects had to be excluded from our main analysis. Four controls were excluded for movement in the fMRI apparatus (>2 mm, see below). The only two patients who were classified as resolved were excluded from analysis because two subjects were not enough to allow any substantial group inferences. The final sample consisted of 11 BPD patients and 17 controls. Exclusion of the six subjects did not affect group homogeneity with respect to age (BPD, 27.8 years ± 6.7; controls, 28.4 years ± 7.5) and education (BPD, 10.8 years ± 1.4; controls, 10.9 years ± 1.6). Comorbidity in the final group included depression (*n* = 6), anxiety or panic disorder (*n* = 2), and somatoform disorder (*n* = 3). Five of the eleven patients were treated with psychotropic medication, including serotonin-reuptake inhibitors (*n* = 2), lithium (*n* = 1) and low doses of neuroleptics (perazin, promethazine and chlorprothixene, *n* = 3). After complete description of the study, participants provided written informed consent. The study was conducted in conformance with the Declaration of Helsinki. The protocol was approved by the local institutional ethics committee of the University of Ulm. Clinical characteristics of the sample are shown in Table 1.

**Table 1:** **Two-group-comparison of clinical scales**.

	C Control (*n* = 16)		B Borderline (*n* = 13)		C×B effect size	Exact U-test	
Clinical scales	*M*	*SD*	*M*	*SD*	*d*	*Z*	*p*
GSI (SCL-90)	**0.22**	0.22	**1.48**	0.51	3.34	4.432	0.000
Barrett impulsivity scale	**67.38**	9.99	**85.23**	10.36	1.75	3.715	0.000
Dissociative experience scale	**4.23**	3.91	**15.66**	16.31	1.02	3.390	0.001

*d, Cohen’s effect size; Z and p, Exact two-tailed Mann-Whitney U-test*.

### Procedure and Measures

#### Attachment Stimulus Presentation and Attachment Coding

**Figure 1:**
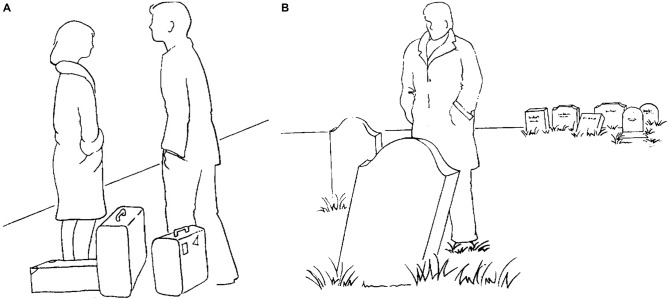
**(A)** Picture “Departure” from the Adult Attachment Projective Picture System (AAP) © (George et al., 1999). **(B)** Picture “Cemetery” from the AAP © (George et al., 1999).

Participants were administered with the fMRI-adapted version of the Adult AAP (Buchheim et al., 2006a), a validated representational adult attachment measure. The measure is comprised of a set of eight drawings, one neutral scene and seven attachment scenes. The picture set includes scenes that depict events associated with attachment activation, such as illness, separation, solitude, death, and threat (Bowlby, 1973). The picture presentation order is designed to gradually increase the activation of the attachment distress (George et al., 1999; George and West, 2003), a methodological feature that has been validated using fMRI analysis (Buchheim et al., 2006b). Pictures are administered in the following sequence: Child at Window—a child looks out a window; Departure—an adult man and woman stand facing each other with suitcases positioned nearby; Bench—a youth sits alone on a bench; Bed—a child and woman sit opposite each other on the child’s bed; Ambulance—a woman and a child watch someone being put on an ambulance stretcher; Cemetery—a man stands by a gravesite head stone; and Child in Corner—a child stands askance in a corner (example picture stimuli are provided in Figure 1).

AAP responses are classified on the basis of verbatim transcribed narratives. The coding system defines unresolved attachment as failure to contain frightening or threatening narrative material demonstrated, for example, by story elements representing attachment dysregulation, such as death, attack, abuse, or devastation (George et al., 1999; George and West, 2003; Buchheim and George, 2011). Resolved attachment is designated when the story material either does not include these elements, or if included, the narrative demonstrates emotional integration or mental organization. This requires the story characters to demonstrate the capacity to think through solutions that describe drawing on internalized attachment resources (the “internalized secure base”), seeking out attachment figures, the capacity for positive and constructive action, or others coming to provide comfort, explanation, or assistance to quell frightening distress (Table 2). Resolved attachment status groups include secure, dismissing, and preoccupied. Secure attachment is characterized by story elements that demonstrate the capacity to think about events and feelings, and expectations for comfort from or mutual enjoyment in attachment relationships. Dismissing attachment is characterized by evidence of physical and psychological distance from attachment figures and distress in story themes, often deflecting needs for care and the desire for attachment figures’ comfort to other activities (e.g., achievement, peer relationship). Preoccupied attachment is characterized by representational confusion; the representation of people or events associated with story themes are diffused by vacillation among different and sometimes opposing ideas.

**Table 2:** **Transcript examples of a “resolved” and two “unresolved” stories to the AAP picture “Cemetery”**.

Resolved AAP story (Control)	Unresolved AAP story (Control)
“An elder man in the graveyard. The man is standing in front of his mother’s tombstone. As he accidentally visited his hometown he also visited the graveyard and lays down a bunch of flowers to his mother. He is thinking about the past, how things had been when she was still alive, what she had pointed out to him for his live. He is very centered upon the past remembering many things and at the same time he is gathering courage for the future since he knows that life is transient. He is keeping to this task for a while; then he returns to his apartment lost in thought. The next day he is leaving his hometown after having visited some of his old friends and some of mother’s neighbors to talk to”.	“A man is standing besides a grave. His wife has recently died. She died suddenly in a car accident. The man is totally in despair. Once a week he is visiting her grave. He finds it difficult to say goodbye. He often talks to her. He communicates with her about whether he is doing things the right way and how hard it is for him to raise his three children all by himself. And how helpless the children are to have lost their mother too early. It’s my impression that he needs a lot of time to come to terms with the situation”.

	**Unresolved AAP story (Borderline patient)**

	“On a graveyard a man is standing by a grave he had been searching for many years. It’s the grave of his parent, who gave their son up for adoption. Their son wants to finally say farewell to his biological parents and he wants to know where his roots are to be found. He is staying on the graveyard for a while, then moves on to an inn and gets drunk until the next evening hoping that in this way he could bury his past just like his parents are buried there. He feels suicidal and like an orphan with no roots. He will never return. He wants to erase the bad past with an adoptive family”.

*Note: Words representing attachment dysregulation are underlined*.

The psychometric properties of the AAP were established in an independent validity study with 144 subjects from a healthy community sample of non-patient subjects. This study demonstrated strong psychometric validity, including meeting psychometric standards for inter-judge reliability, test-retest reliability (after 3 months), discriminant validity, and convergent construct validity with another validated developmental attachment narrative assessment, the AAI (George et al., 1984/1985/1996; George and West, 2003, 2012).

In the present study, two blind, reliable AAP judges independently coded the AAP narratives of the stories that participants told in the scanner. There was unresolved-resolved agreement in 27 out of 28 cases (96%). The resulting inter-rater agreement was κ = 0.93, corresponding to “almost perfect agreement” by Landis and Koch (1977). AAP validity for scanner-produced stories was established through convergent classifications with the AAI, which were administered 1 month after fMRI AAP acquisition and classified by a blind trained reliable AAI judge. As also reported in a review on AAP classifications in different clinical groups (Buchheim and George, 2011), the correspondence between the AAP and AAI resolved vs. unresolved categories in this sample was κ = 0.70, corresponding to “substantial agreement” by Landis and Koch (1977).

#### MRI Acquisition

##### Experimental procedure

Subjects were instructed in the AAP story telling task before they entered the scanner using two non-AAP “neutral” (i.e., not attachment related scenes) pictures. The pictures were same size and drawn in the same style as the stimuli in the AAP set. The goal was for subjects to understand the scope of the probes normally asked during in-person administration. The training procedure was repeated two more times, as needed to achieve this goal. The fMRI acquisition started with the original AAP picture presentation and instructions. During scanning, subjects were visually presented the standard AAP instruction at the introduction of the stimulus (“what led up to that scene, what are the characters thinking or feeling, and what might happen next?”) for 10 s and a fixation cross for 10 s. Afterwards each AAP picture was presented (120 s) along the original order of the measure (described in the “Materials and Methods” Section). Subjects were asked to talk about the picture for at least 2 min. A fixation cross was shown for 15 s after the picture presentation until beginning a new cycle of instruction and the next picture presentation. The total procedure included nine pictures, the two neutral and seven standard AAP attachment scenes.

##### Data acquisition

1.5 Tesla Siemens Magnetom Symphony scanner (Siemens, Erlangen, Germany), image size: 64 × 64 voxels, FOV of 192 mm, slice thickness 4 mm with 1 mm gap, 25 slices covering the whole brain, TE/TR 40 ms/2500 ms, total acquisition time 25 min (=598 volumes, one session). Instructions and pictures were shown with fMRI compatible video-goggles (Resonance Technologies, Northridge, CA, USA). Speech was digitally recorded beginning at the onset of each picture using an fMRI compatible microphone and saved digitally on a computer using Cool Edit Pro (Syntrillium Software Cop. Phoenix, Arizona). Head movement was minimized by using padded earphones fixating the head within the gradient insert coil.

#### Statistical Analysis and Image Analysis

Group differences of the behavioral attachment data were analyzed using the exact Mann-Whitney U-test and the Kruskal-Wallis H-test and (SPSS version 14). We used non-parametric tests because of the non-normal distribution of the dependent variables. Preprocessing and statistical analysis of fMRI data were carried out with SPM2^2^ and MATLAB 6.1 (MathWorks, Natick, MA, USA). The first four functional images were discarded to account for equilibration effects. Individual functional images were corrected for motion artifacts by realignment to the first volume of each session. As noted earlier, we excluded four control subjects because of excessive head movement (>2 mm within a trial cycle) in order to minimize movement effects. Further preprocessing included spatial normalization (3 × 3 × 3 mm) and smoothing (FWHM 8 mm). The regression model for each subject was as follows: each of the nine pictures had three or two individual regressors with variable duration depending on the time of speech: Regressor 1, modeling the time from onset of picture till onset of speech; regressor 2, modeling the picture during speaking; and regressor 3, modeling the time from offset of speech till end of picture presentation (if the subject did talk for less than 2 min). Three more regressors were built, each modeling all nine pictures: Regressor 4, (onset of every single word of all pictures as a stick function); regressor 5, (instructions); and regressor 6, (all fixation crosses = base line). Regressors of interest were convolved with a function that modeled a prototypical hemodynamic response before inclusion into the regression model. A high-pass filter was set at a cutoff frequency of 240 s. Finally, six more regressors modeled residual motion. For each trial the variance of each voxel was estimated according to the General Linear Model Individual regionally specific effects of interest were calculated for each participant using linear contrasts, resulting in a *t*-statistic for every voxel.

The effects of interests in this study were narrative story responses to the seven attachment pictures. We calculated the contrast picture presentation for each subject during speech (regressor 2) + picture presentation before the subjects start to speak (regressor 1) vs. baseline (fixation cross), thereby including potential mental processes before the actual speaking phase starts. For each subject, contrasts for single pictures were calculated, that is, seven contrasts for the attachment pictures ordered 1–7.

A within subject repeated measures ANOVA with three groups (resolved healthy controls, unresolved healthy controls and unresolved BPD patients) and seven repeated measurements was calculated as a second level analysis in order to test for effects of group and attachment classification. The AAP is designed to activate the attachment system increasingly from picture 1–7. The contrast of interest within each group was labeled “AAP effect” (–3 –2 –1 0 1 2 3), in accordance with the results of our pilot study (Buchheim et al., 2006a). *t*-statistics for each voxel were set at a threshold of *p* < 0.001 uncorrected for multiple comparisons and a cluster threshold of *p* < 0.05. We allowed a small volume correction for regions of interest with family wise error correction of *p* < 0.05 for those regions for which we had* a priori* hypotheses (amygdala, DLPFC, dorsal anterior cingulate) using a radial sphere of 10 mm (amygdala) and 20 mm (DLPFC, dACC), respectively. Talairach and Tournoux (1988) and Duvernoy (1999) atlases were used to identify all brain areas.

## Results

### Behavioral Data

Borderline patients differed significantly from controls in all clinical scales (Table 1). As expected, the majority of the BPD patients were judged unresolved (11/13, 85%) in the AAP. Ten of seventeen (59%) controls were judged resolved and seven (41%) were judged as unresolved. In the fMRI data analysis, we included 11 patients (all coded as unresolved) and 17 controls (*n* = 7 unresolved, *n* = 10 organized: *n* = 6 secure, *n* = 4 dismissing, no preoccupied subjects). The overall distribution of attachment status differed significantly between borderline patients and controls (Fisher’s exact test, *p* = 0.026).

### Neuroimaging Data

Both control and patient groups showed activations in bilateral occipital cortex, bilateral superior medial frontal cortex, bilateral precentral and left inferior frontal gyrus for the main effect of picture presentation and bilateral medial and superior temporal gyrus, bilateral precentral gyrus, medial occipital gyrus and cerebellum for the main effect of speech production.

**Figure 2:**
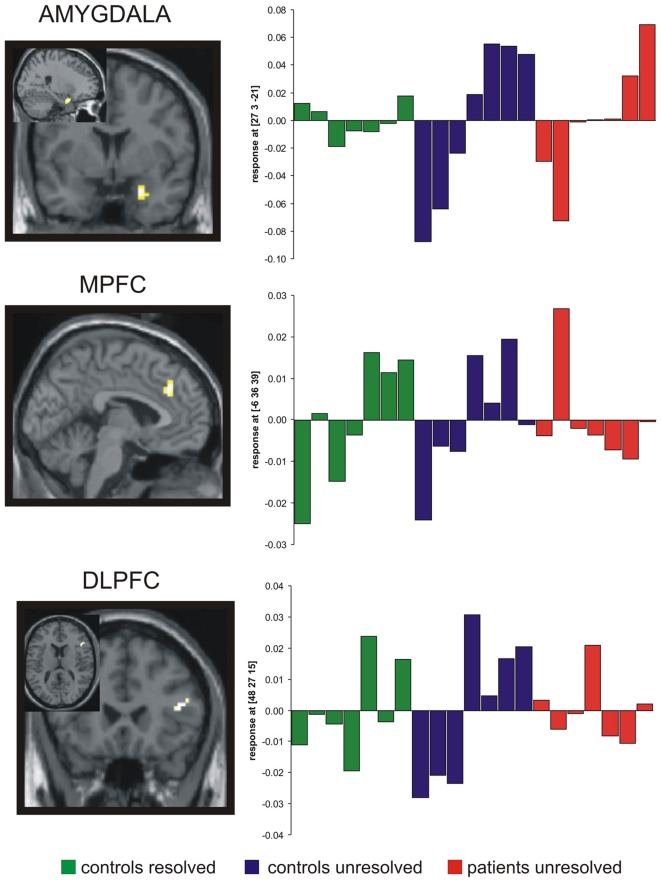
**Results of the functional magnetic resonance imaging (fMRI) analysis.** A significant AAP effect (increasing activation along the task) was found in the amygdala for both, unresolved controls and patients, but not in the resolved controls. A significant AAP effect in the medial PFC was observed in both control groups, whereas the dorsolateral prefrontal cortex (DLPFC) exhibits an AAP effect only in the group of unresolved controls. All regions were significant in the respective interaction analyses (see Table 3). Images of statistic parametric mapping are projected onto sections of the standard T1 template of SPM 2. Plots of contrast estimates for each condition were shown on the right. Green bars, resolved controls; blue bars, unresolved controls; red bars, unresolved patients.

The goal of the neuroimaging analysis was to examine the AAP effect (increasing attachment activation during the task) in control and borderline subjects. The first analysis examined the AAP effect for the control group. Three regions showed increasing activation during the task: right amygdala, right DLPFC and mPFC in the rostral cingulate zone (RCZ; Table 3). The second analysis compared all controls with all patients. We found a significantly stronger activation of the RCZ in the healthy controls and a significantly stronger activation of the right amygdala in the patient group. The third analysis examined these three regions in order to understand the contribution of attachment status and diagnosis to these activations. This analysis was performed by contrasting the three subgroups of our sample with each other (Figure 2).

**Table 3:** **fMRI results**.

Region	BA		*Z*	*x*	*y*	*z*
*all CTRLs*
Amygdala		R	4.51	24	−3	−27
Dorsolateral prefrontal cortex	46	R	3.54	48	24	15
Medial prefrontal cortex	9		4.09	−3	33	33
*CTRLs resolved*
Superior temporal sulcus	39	L	4.24	−51	−60	21
*CTRLs unresolved*
Amygdala		R	4.60	24	3	−27
Dorsolateral prefrontal cortex	46	R	3.71	48	27	15
Superior frontal gyrus	6	R	4.26	21	15	63
*all unresolved (CTRLs and PATs)*
Amygdala		R	4.42	24	3	−24
Superior temporal sulcus	39	R	3.87	57	−51	12
Medial prefrontal cortex	8	R	3.85	3	21	45
*PATs unresolved*
Amygdala		R	3.66	21	−6	−21
Anterior cingulate cortex	32	R	3.70	3	21	33
Cingulate gyrus	24	R	3.58	3	−18	42
Superior temporal sulcus	39	R	3.99	60	−57	15
*all CTRLs > PATs unresolved*
Medial prefrontal cortex	8	L	2.92*^,#^	−6	36	39
*CTRLs resolved > PATs unresolved*
Medial prefrontal cortex	8	L	2.83*	−6	36	39
*all unresolved (CTRLs and PATs) > CTRLs resolved*
Amygdala		R	3.15*^,#^	27	3	−21
*CTRLs unresolved > CTRLs resolved*
Amygdala		R	3.41*^,#^	27	3	−24
Dorsolateral prefrontal cortex	46	R	2.51*	48	27	15
*PATs unresolved > CTRLs resolved*
Amygdala		R	2.88*^,#^	21	−6	−21
*CTRLs unresolved > PATs unresolved*
Dorsolateral prefrontal cortex	46	R	3.11*^,#^	48	24	15
Medial prefrontal cortex	8	L	2.44*	−6	36	39

All results: p < 0.001 uncorrected for multiple comparisons at voxel level, p < 0.05 at cluster level; *p < 0.05 corrected for small volume and ^#^additionally corrected for number of volumes of interest (n = 6); BA, Brodmann area; x, y, z, respective coordinates of MNI template.

The results of these analyses are shown in Table 3. An AAP effect was found in the amygdala in both unresolved groups (unresolved controls and unresolved BPD patients). An AAP effect in the RCZ was found only in the controls (resolved and unresolved). An AAP effect in the right DLPFC was found only in the unresolved controls.

In order to check that our results are not solely due to *medication* effects, we performed an additional analysis including only patients taking no psychotropic drugs (*n* = 6). Results were the same: all CTRLs > unmedicated PATs unresolved: mPFC (−9, 36, −39, *Z* = 3.63); CTRLs resolved > unmedicated PATs unresolved: mPFC (−9, 36, −39, *Z* = 3.54); unmedicated PATs unresolved > CTRLs resolved: Amygdala (21, −6, −21, *Z* = 3.39); CTRLs unresolved > unmedicated PATs unresolved: DLPFC (48, 24, 15, *z* = 2.53).

## Discussion

The present study examined the neural correlates of attachment dysregulation in a group of BPD patients compared to controls. This study used a paradigm that evaluated neural response patterns while subjects told attachment stories in response to AAP stimuli in the fMRI scanner. The fMRI analysis model followed the logic of the design of the attachment measure. According to this logic, the picture presentation sequence increasingly activates the attachment distress. We labeled this increasing activation over the course of task as “AAP effect.” Due to the fact that almost all BDP patients were classified as unresolved, we investigated only three classification groups: resolved controls, unresolved controls and unresolved BDP patients. There were three main imaging finding. First, all unresolved subjects (borderline and controls), but not the resolved controls, showed the AAP effect reflected in an increasing amygdala activation. Second, all controls (resolved and unresolved), but not the unresolved patients, showed the AAP effect in relation to increasing activation of the RCZ. Third, only the unresolved controls showed an AAP effect of increasing activation of the right DLPFC. This effect was not found in the resolved controls or the unresolved patients. We now discuss these results in the context of attachment research.

The predominant unresolved classification in the BPD patients was consistent with previous research (Fonagy et al., 2000; Agrawal et al., 2004; Bakermans-Kranenburg and van IJzendoorn, 2009; Buchheim and George, 2011). This was found using two independent attachment measures (AAI and AAP). How are these attachment findings related to the neural patterns found in the three groups (unresolved patients, unresolved and resolved controls)?

As shown in Figure 2, the AAP effect was present in the right amygdala in both unresolved groups. This finding strengthens the results of our pilot study with healthy subjects (Buchheim et al., 2006a). Amygdala activation has been found in other studies to be associated with a range of negative and positive emotional stimuli (Davis and Whalen, 2001). A recent study by White et al. (2014) demonstrated that the amygdala is responsive to animate and emotional stimuli. Moreover, the authors consider that the interaction between the various functions of the amygdala may need to be taken into account simultaneously to fully understand how they interact. Although amygdala activity has been suggested to be modulated by affective and non-affective factors, there is considerable controversy regarding its specific functional nature. Costafreda et al. (2008) examined the effects of experimental characteristics on the probability of detecting amygdala activity in a meta-analysis of 385 functional neuroimaging studies of emotional processing. The authors reported that all emotional stimuli were related to higher probability of amygdala activity than neutral stimuli. Comparable effects were detected for most negative and positive emotions, however there was a higher probability of activation for fear and disgust as compared to happiness (Costafreda et al., 2008). The stimuli used in the present study were not general emotional stimuli. The AAP picture stimuli were selected specifically because of their ability to activate attachment distress by introducing themes like, e.g., separation, loneliness, danger or loss. Therefore, we interpret this amygdala activation as a neural correlate of negative emotional arousal that is associated with dysregulated attachment fear that is evident in the unresolved verbatim narratives. In contrast, resolved controls do not show the AAP effect in the amygdala. Resolved attachment is defined by the ability to re-organize attachment related fearful and threatening themes emerging in their stories. This re-organization appears to have blocked the AAP effect in the amygdala. Attachment activation of resolved controls, therefore, is not dysregulated by the negative emotions associated with distress.

Increased amygdala activation in BPD patients has been found in a variety of passive stimulation paradigms and these findings are interpreted as heightened emotional sensitivity to aversive stimuli (Davis and Whalen, 2001; Herpertz et al., 2001; Donegan et al., 2003; Silbersweig et al., 2007; Hazlett et al., 2012; Kamphausen et al., 2013; Schulze et al., 2016). Our results are consistent with these findings. Moreover, they provide an empirical link to the frequently reported unresolved attachment found in BPD samples, as mentioned before (Agrawal et al., 2004; Bakermans-Kranenburg and van IJzendoorn, 2009; Buchheim and George, 2011). As the amygdala activation AAP effect was found in both our BPD and unresolved controls, the effect is likely to be related to adverse early experiences that are not necessarily specific to BPD diagnosis. In order to disentangle the contribution of attachment and diagnosis, it would be desirable to include a resolved BPD group. This was not possible in the current study because the small number of resolved BDP patients precluded such an analysis.

The RCZ showed the AAP effect in both control groups, resolved or unresolved. Studies in humans showed that the RCZ (the posterior MFC border zone between the medial areas BA8, BA6 and BA32’ with some extension into BA24’) is involved in monitoring for unfavorable outcomes, performance and conflict monitoring, and decision uncertainty (Carter et al., 1998; Botvinick et al., 1999; Ridderinkhof et al., 2004; Fan et al., 2008; Nee et al., 2011). A recent comprehensive review discussed the diverse functions of the RCZ and proposed that this diversity can be understood in terms of the allocation of control based on monitoring the expected value of control (Shenhav et al., 2013). Although a subgroup of the healthy participants showed the failure to reorganize their attachment distress in their narratives on a behavioral level (unresolved), they demonstrated the ability to control their distress on a neural level. As the attachment stories in our study include distressing and potentially threatening themes, one possible interpretation of this activation is that all control subjects monitored their stories for unfavorable outcomes, that is, how the story might continue (part of the instruction). This monitoring function seems to be impaired in the BPD patients of our sample. An alternative interpretation is that activation in this region simply reflects emotional involvement. However, this is unlikely given that the three groups showed a different pattern.

The AAP effect in the DLPFC was found only in the unresolved control group; it was not observed in the resolved controls or the unresolved patients. The right DLPFC has been described as being involved in cognitive control (Duncan and Owen, 2000), emotion regulation (Ochsner and Gross, 2005), and more generally in executive functions, regulatory mechanisms that help individuals cope with extraordinary affordances. Recent research findings suggested a regulatory hierarchy, whereby the DLPFC and areas of the anterior medio-PFC modulate the cingulate, which in turn modulates the amygdala and further subcortical areas (Meyer-Lindenberg et al., 2005; Buckholtz et al., 2007). Several neuroimaging studies described a dysfunction of the right DLPFC in BPD patients (De La Fuente et al., 1997; Soloff et al., 2000; Schmahl et al., 2003). One recent study reported that cortical thickness in the DLPFC of female BPD patients without PTSD correlated positively with emotion regulation scores, which was also positively associated with amygdala volume, as well as cortical thickness of the insula (Bruehl et al., 2013). These findings suggested possible compensatory mechanisms for the impaired emotion regulation. Resolved subjects in the present study demonstrated a low incidence of threatening situations in their narratives, suggesting that the cognitive control system was not increasingly engaged over the course of the task. Unresolved controls, however, had increasing affective involvement. We, therefore, interpret the accompanying right DLPFC activation as an effort in this group to cope with this increasing affective involvement.

BPD patients were showing the highest proportion of unresolved attachment patterns in this sample. The fMRI findings suggest that they were neither able to recruit the DLPFC (cognitive control) nor the RCZ (conflict monitoring) while being emotionally overwhelmed, as reflected in the enhanced amygdala activation. However this finding contrasts with some published neuroimaging studies of affective dysregulation in BPD, which used passive stimulation. Studies on functional correlates of response inhibition have demonstrated further evidence for functional impairments of prefrontal areas, particularly of the DLPFC, the rostral ACC, and the orbitofrontal cortex (OFC). Minzenberg et al. (2007) used for example an implicit affect regulation task (responses to threatening vs. neutral faces) and found specifically enhanced neural activation of the right amygdala in BPD along with attenuated activations of the rostral ACC. Although our interpretation of the different activation patterns shown in Figure 2 cannot be directly proven, we suggest that we provided a novel and interesting approach to understanding emotional instability in BPD patients with respect to attachment on a neural basis. The participants in our task were actively talking about their subjective perception of attachment scenes reflecting their individual mental organization of these crucial topics in relation to their history of abuse, maltreatment and emotional neglect.

### Limitations

There are several limitations to our study that need to be taken into account when interpreting our findings. First, the number of resolved BPD patients (*n* = 2) was too small to include in our analysis and moreover we were not able to recruit a larger sample of BPD patients willing to participate in such a demanding paradigm (talking in the fMRI scanner), which limits the generalization of our results. The attachment literature suggests, that unresolved attachment is the predominant classification group, therefore future research would require a substantially large sample in order to include resolved BPD subjects. Although this limits the interpretation of our data to a certain extent, we were able to meaningfully evaluate the results using the three-group designations afforded by the participant attachment distribution in the current study.

Second, we did not include a clinical control group. This leads to questions as to whether our results may also be present in patients with other psychiatric disorders and not specific to BPD.

Third, 5 out of 11 of the BPD subjects were under low dose medication. This could be a confounding factor in comparing patients and controls, although our control analyses of medication-free patients only speaks against this assumption.

Fourth, this study aimed to characterize differences between healthy participants and BPD patients using an attachment paradigm along the AAP system focusing on attachment related pictures only. Since this study has identified differences between controls and patients, it is rather difficult to interpret the data univocally in the absence of an adequate control stimulus set, which is the focus of a recent study with healthy controls (Labek et al., 2016). Moreover, it would have been appropriate to add an established emotion-inducing paradigm for comparing neural responses to attachment plus to emotional involvement and/or emotional regulation capacities. Strictly spoken without this contrasting paradigm we may not directly conclude that we have investigated emotional dysregulation in BPD patients compared to controls.

Fifth, all results were derived from the three group analyses. However, this might have inflated statistical results. Therefore, we also calculated separate analyses for those contrasts where only two groups were involved. All results remain significant, showing that increased degrees of freedom did not generally inflate our results. Only mPFC activation in the contrast comparing resolved controls and unresolved patients does not surpass additional small volume correction.

Finally, overt speech in the scanner always is accompanied with head movements. The head movement in this study was less than 2 mm, and we took steps to eliminate residual influences. These included dropping subjects, including movement parameters as a covariate of no interest, and modeling the onset of every spoken word.

### Conclusions

Unresolved controls, but not patients, showed activation in the right DLPFC and the RCZ. We interpreted this as a neural signature of BPD patients’ inability to exert top-down control under conditions of attachment distress. These findings point to possible neural mechanisms for underlying affective dysregulation in BPD in the context of attachment trauma and fear. We found that both increased emotional sensitivity, as well as impaired emotion regulation, may have contributed to affective dysregulation in unresolved BPD patients. Increased emotional sensitivity, as evidenced by an AAP effect in the right amygdala, might be explained in large part by their attachment pattern. An alteration of the cognitive control system (RCZ and right DLPFC) is found when unresolved attachment and diagnosis of BDP are present simultaneously. Modulation of BPD patients’ responses in attachment situations during treatment and psychotherapy to patterns similar to those described in the control group might be an important indication of their increasing capacity to regulate attachment distress and to show sufficient cognitive control (Perez et al., 2016). Future studies may examine as to what extent psychotherapy has the potential to change brain activation from a more unresolved to a more resolved pattern.

## Author Contributions

The study was conceptualized by HW, AB, SE, CG, HK and MS. The study setup and data collection were organized and conducted by HW, AB, SE, MS, HK and PM. fMRI analyses was performed by HW and SE. Coding of attachment interviews were conducted by AB and CG. SE, HW and DP performed the statistical data analysis and contributed substantially to the result interpretation. HW, AB, SE, CG, and DP provided important intellectual contribution in commenting and revising the manuscript. AB, HW, SE, and CG wrote the manuscript and edited its final version.

## Conflict of Interest Statement

The authors declare that the research was conducted in the absence of any commercial or financial relationships that could be construed as a potential conflict of interest.

## Footnotes

^1^PM has been trained and certified for reliable diagnosis in the SCID-rating by Prof. Wittchen, Munich, Germany and by A. Loranger MD, New York, NY, USA regarding IPDE. In seven patients an experienced Master’s level psychologist conducted a second SCID interview. Agreement between the raters was κ = 1.0 for both BPD and lifetime depressive episode.

^2^http://www.fil.ion.ucl.ac.uk

## Abbreviations

AAI: Adult Attachment Interview
ACC: anterior cingulate cortex
AAP: Adult Attachment Projective Picture System
BA: Brodmann area
BPD: Borderline personality disorder
CTR: controls
DLPFC: dorsolateral prefrontal cortex
IPDE: International Personality Disorder Examination
mPFC: medial prefrontal cortex
OFC: orbitofrontal cortex
PAT: patients
PFC: prefrontal cortex
PTSD: posttraumatic stress disorder
RCZ: rostral cingulate zone
SCID-I: Structured Clinical Interview for DSM-IV.
